# Exploring the Antimicrobial Efficacy of Graphene Oxide: Key Mechanisms and Future Directions

**DOI:** 10.3390/pharmaceutics18080966

**Published:** 2026-08-06

**Authors:** Yuezi Li, Zhihong Ke, Bilan Deng, Ying Chen, Xundong Lin, Aijie Chen, Weihong Guo, Xiaoli Feng

**Affiliations:** 1 Stomatology Hospital, School of Stomatology, Southern Medical University, Guangzhou 510515, China; liyzi1026@163.com (Y.L.); 15914303482@163.com (B.D.); chenaijie@smu.edu.cn (A.C.); 2 Department of General Surgery, Nanfang Hospital, Southern Medical University, Guangzhou 510515, China; kezhihong2025@163.com (Z.K.); cyyyy1203@163.com (Y.C.); drap0824@163.com (X.L.)

**Keywords:** graphene oxide, oxidative stress, photothermal therapy, antibiotic-resistant bacteria, caries prevention, antibacterial dressings, gastrointestinal infection

## Abstract

The inappropriate utilization of traditional antimicrobials has expedited the development of multidrug-resistant bacterial strains, thereby generating an urgent demand for innovative, safe, and efficacious alternatives. Graphene oxide (GO), characterized by its distinctive physicochemical properties, holds significant potential for a wide range of biomedical applications, such as the prevention of oral diseases, periodontal therapy, control of gastrointestinal infections, wound healing, antibacterial therapy for the respiratory tract, and management of urogenital infections. The antimicrobial mechanisms of GO include physical disruption of microbial membranes, induction of reactive oxygen species (ROS), and its role as a photosensitizer. Moreover, improving the dispersion, stability, release behavior, and reusability of GO-based composites can markedly enhance their effectiveness. The antimicrobial activity of GO is further modulated by its physicochemical properties, synthesis methods, bacterial characteristics, and environmental conditions, while potential synergistic or antagonistic interactions remain to be elucidated. This review synthesizes recent advancements in GO-based antimicrobial strategies, elucidates key mechanisms and influencing factors, and addresses major challenges and future research directions, with the objective of facilitating the clinical translation of GO as a next-generation antimicrobial material.

## 1. Introduction

The misuse and overuse of conventional antimicrobials have accelerated multidrug resistance across various bacterial species. Beyond resistance, certain antimicrobial substances pose significant health and environmental risks. For instance, silver nanoparticles (AgNPs) are widely utilized for their broad-spectrum efficacy against bacteria, fungi, and viruses. However, growing evidence highlights their potential toxicity to public health and the ecosystem [1]. These challenges necessitate the development of innovative antimicrobial materials that prioritize both high efficacy and biological safety.

With the rapid advancement of nanomaterials, graphene oxide (GO) has attracted considerable attention in biomedicine owing to its unique two-dimensional layered structure and excellent physicochemical properties. GO is typically produced through the oxidation and subsequent exfoliation of graphite, yielding flexible, two-dimensional nanosheets with a thickness of approximately 1 nm. Its structural comprises sp^2^-hybridized graphitic domains and sp^3^-hybridized oxidized domains, together with structural features such as nanoscale pores [2]. According to the widely accepted Lerf–Klinowski model, epoxy (C–O–C) and hydroxyl (–OH) groups are predominantly located on the basal plane, whereas smaller amounts of carbonyl (C=O) and carboxyl (–COOH) groups are mainly located at the sheet edges. This distinction in chemical environment between the basal plane and edges provides abundant reactive sites for the controlled functionalization of GO and the fabrication of GO-based composites [3].

The layered structure and surface chemistry of GO endow it with a range of favorable physicochemical properties. Its two-dimensional nanosheet architecture offers a large specific surface area and abundant binding sites for the loading of therapeutic agents, metallic nanoparticles, and bioactive molecules [4,5]. In addition, the abundant oxygen-containing functional groups on the GO surface, including carboxyl, hydroxyl, and epoxy groups, permit covalent functionalization through reactions such as amidation, esterification, and epoxy ring-opening, as well as functionalization through noncovalent interactions, including π–π stacking, hydrogen bonding, and electrostatic interactions [6]. GO exhibits an amphiphilic character, arising from the coexistence of. Hydrophilic oxygen-containing functional groups and a hydrophobic sp^2^-hybridized graphitic domains. This dual character supports its dispersion in aqueous media under appropriate conditions and facilitates effective interactions with bacterial cell membranes and various nanomaterials, thereby providing a versatile platform for constructing multifunctional antibacterial composites [7]. These physicochemical characteristics also distinguish GO from many existing antibacterial nanomaterials. Compared with polymeric nanocarriers, which mainly serve as drug-delivery vehicles, GO possesses intrinsic antibacterial activity while simultaneously providing abundant oxygen-containing functional groups for further functionalization and controlled drug release [8]. Furthermore, unlike pure metal nanoparticles, GO-based metal nanocomposites exhibit improved stability and more uniform nanoparticle distribution while integrating the intrinsic antibacterial activity of GO with the antimicrobial properties of metallic nanoparticles, thereby enabling synergistic antibacterial effects [9,10].

The intrinsic antibacterial activity of GO is mediated through multiple complementary mechanisms. For example, GO’s sharp edges can physically disrupt cell structures, causing the leakage of intracellular contents [11]. Additionally, GO may induces reactive oxygen species (ROS) generation, leading to oxidative stress, lipid peroxidation, and cell death [12]. The surface functional groups also facilitate efficient interactions with biomolecules (e.g., antibiotics) or nanomaterials (e.g., metal nanoparticles). These interactions form composites with synergistic antibacterial effects, expanding their potential in antimicrobial research [13,14].

Currently, GO and its composites show significant promise in various biomedical applications, such as treating oral and gastrointestinal infections, wound healing, and respiratory or urogenital protection. In dental therapy, GO nanosheets inhibit *Streptococcus mutans* (*S. mutans*) and *Porphyromonas gingivalis* (*P. gingivalis*) by disrupting cell walls and membranes [15]. GO and its composites also exhibit cytotoxicity against gastrointestinal pathogens like *Escherichia coli* (*E. coli*) and *Staphylococcus aureus* (*S. aureus*) [16,17]. Furthermore, GO effectively eradicates *Klebsiella pneumoniae* (*K. pneumoniae*) and *Pseudomonas aeruginosa* (*P. aeruginosa*), suppressing infection spread and reducing tissue inflammation in murine models [18]. In addition, a 2025 study reported the design of a composite hydrogel by integrating GO with AgNPs, which functioned as a novel wound-dressing material with potent antibacterial efficacy [13]. Consequently, GO remains a premier candidate for biofunctionalization and exhibits tremendous potential for antimicrobial applications.

Nevertheless, the intricate nature of its antibacterial mechanisms, the multifactorial determinants of its efficacy, and practical constraints limit its widespread adoption. Future research must thoroughly elucidate GO’s antibacterial mechanisms and delineate the factors influencing its activity to enhance clinical translation. This review systematically synthesizes recent advancements in GO-based antibacterial research, emphasizing mechanisms of action, composite applications, and clinical challenges (illustrated in the graphical abstract). We aim to provide theoretical insights and novel perspectives for developing effective and safe GO-based antimicrobial materials.

## 2. Antimicrobial Applications of GO and Its Composite Materials

### 2.1. Oral Applications

Currently, GO and its composite materials have found increasing application in oral healthcare, particularly in the prevention of dental caries and management of periodontal and endodontic diseases [19,20]. *S. mutans* is widely recognized as the principal etiological agent of caries development, and its ability to establish resilient biofilms poses a significant therapeutic challenge [21]. The incorporation of GO nanoparticles into mouth rinses has been shown to enhance bactericidal activity against *S. mutans* without diminishing enamel hardness or eliciting adverse effects on the oral mucosa [22]. Moreover, GO not only suppress biofilm formation but also promote the remineralisation of casein phosphopeptide-amorphous calcium phosphate (CPP-ACP) under salivary conditions while maintaining excellent biocompatibility [23].

Anaerobic and facultative anaerobic bacteria, such as *P. gingivalis* and *Actinobacillus* species, are recognized as the primary pathogens of periodontitis. GO nanosheets have also been shown to compromise the structural integrity of the cell walls and membranes of *P. gingivalis* and *Fusobacterium* species, thereby suppressing their viability in a dose-dependent manner [12]. In addition, GO can serve as a versatile carrier for antibiotics, such as minocycline hydrochloride or nanosilver ions (Ag^+^), exhibiting pronounced synergistic bactericidal effects against Gram-positive *S. aureus* and *S. mutans* [24,25].

The persistence of pathogenic bacteria within the root canal system is the principal cause of endodontic treatment failure, and the Gram-positive bacterium *Enterococcus faecalis* (*E. faecalis*) is the predominant etiological agent [26]. Calcium phosphate bone cement (CPC) is frequently used as a root canal sealer. Recent studies have developed an injectable CPC-chitosan paste incorporating GO, which exerts antibacterial activity by disrupting *E. faecalis* biofilms. This composite material has shown potential to enhance the success rate of root canal therapy [27].

### 2.2. Gastrointestinal Applications

To date, no studies have reported the therapeutic potential of GO in *Helicobacter pylori* infections. Consequently, this section focuses on the applications of GO and its composites against intestinal microbiota, particularly opportunistic pathogens such as *E. coli* and *S. aureus*.

Enterotoxigenic *E. coli* (ETEC) is a major pathogen responsible for infectious diarrhea. GO nanosheets exert dose- and time-dependent antibacterial effects against *E. coli* through their intrinsic physicochemical properties [28,29]. Graphene oxide nanoribbons (GONRs) can similarly induce oxidative stress, damaging the lipid components of the bacterial membrane and thereby inhibiting bacterial proliferation [16]. In addition, GO functionalized through thionyl chloride activation and covalent amine modification demonstrated significant antibiofilm activity against *E. coli* [30]. *S. aureus* is another prevalent intestinal pathogen implicated in diseases such as pseudomembranous enterocolitis and antibiotic-associated colitis. Consistent with its antibacterial activity against *E. coli*, GO nanosheets displayed dose-dependent efficacy against methicillin-resistant *S. aureus* (MRSA), maintaining excellent antimicrobial performance even at concentrations as low as 5 μg/mL [31].

### 2.3. Cutaneous Applications

The skin is the body’s primary protective barrier. When applied to cutaneous wounds, antibacterial dressings offer mechanical protection, absorb exudates, and support the preservation of a stable microenvironment surrounding the wound. They also provide low toxicity, ease of use, and antimicrobial efficacy, making them a better approach to treating skin injuries [32]. GO is commonly added to hydrogels, composite membranes, and patches for use as antibacterial dressings because of its advantageous physicochemical characteristics.

Based on the intrinsic antibacterial mechanisms of GO, it can be incorporated as a core antibacterial component into a hydrogel system constructed from polyvinyl alcohol (PVA) and aloe vera extract (Av), which significantly enhances its tensile strength to 1.5 MPa [33]. In addition to its inherent antibacterial properties, GO can be combined with metallic nanomaterials and integrated into wound dressings to achieve synergistic antimicrobial effects. Researchers integrated GO with copper nanoparticles (Cu NPs), which share analogous antibacterial mechanisms. This combination synergistically enhances the production of ROS, leading to the disruption of bacterial cell membranes and DNA. Additionally, it stimulates the production of nitric oxide (NO), which subsequently promotes the expression of vascular endothelial growth factor (VEGF) and facilitates wound healing through angiogenesis [34]. Furthermore, Ag/Cu/GO composite nanomaterials synthesized via chemical reduction have been shown to effectively eradicated *P. aeruginosa* biofilms at concentrations non-toxic to human dermal fibroblasts, offering a promising therapeutic approach for chronic infections, such as diabetic ulcers, pressure injuries, and venous stasis ulcers [35,36].

GO exhibits antibacterial activity in the PVA-CMT-GO composite membrane by inducing oxidative stress, improving the membrane’s mechanical characteristics, and adsorbing drugs. These mechanisms collectively enable the composite membrane, when applied to cutaneous wounds, to exhibit enhanced antibacterial efficacy while simultaneously promoting wound healing [37]. In addition to increasing antimicrobial efficacy, the incorporation of GO lowers the risk of systemic toxicity and bacterial resistance to antibiotics. Furthermore, GO functionalized with the broad-spectrum antimicrobial agent polyhexamethylene guanidine hydrochloride (PHMG) overcomes the limitations of poor dispersibility and stability in hydrogel matrices, enabling the fabrication of skin-like thermoplastic polyurethane (TPU) bilayer dressings with durable and broad-spectrum antibacterial properties [38]. Additionally, an Ag/GO-GelMA-based nanophotothermal platform was created by combining the effective photothermal conversion capacity of Ag/GO nanoparticles with the sustained-release properties of GelMA hydrogels. This platform demonstrated exceptional antibacterial performance and the capacity to speed up the healing of infected wounds [39].

### 2.4. Respiratory Applications

The respiratory tract is in constant contact with the external environment and harbors a diverse community of colonizing microorganisms. Common pathogenic species include *K. pneumoniae*, *P. aeruginosa*, and *Acinetobacter baumannii* (*A. baumannii*).

*K. pneumoniae* is a Gram-negative bacterium that can cause severe respiratory diseases, such as pneumonia and bronchitis [40]. Owing to its resistance to multiple antibiotics including penicillin, *K. pneumoniae* is prone to widespread transmission in hospital environments [41,42]. Stable aqueous suspensions of GO have been shown to exert bactericidal effects against *K. pneumoniae* through physical cutting, thereby reducing its infectivity in the lung tissue of C57BL/6J mice, alleviating pulmonary inflammation, and extending survival [18]. In addition, composite materials combining GO with conventional antimicrobial agents have demonstrated pronounced antibacterial efficacy. For example, in GO-iodine (GO-I) complexes, GO enhances antibacterial activity by anchoring larger quantities of iodine ions, resulting in superior activity against *K. pneumoniae* compared to conventional povidone-iodine (PVP-I) disinfectants [14]. *P. aeruginosa* carries a diverse repertoire of antibiotic-resistance determinants and is a major cause of chronic lung infections in patients with cystic fibrosis. Unmodified GO nanosheets have demonstrated antibacterial activity against *P. aeruginosa* through their intrinsic physicochemical properties [43]. Further studies have revealed that partial fragmentation of GO nanosheets occurs when they are combined with metallic nanoparticles, such as silver (Ag) nanoparticles. This increases the number of sharp edges oriented at multiple angles and facilitates a dense distribution of nanoparticles, thereby enhancing the cutting effect and significantly reducing *P. aeruginosa* viability [44]. *A. baumannii* is another clinically significant multidrug-resistant pathogen frequently associated with hospital-acquired infections. GO-Ag nanocomposites have been reported to exhibit strong antibacterial activity against *A. baumannii* as well as *E. coli* in hospital settings. The antibacterial mechanism is attributed to a combination of physical membrane disruption, bacterial entrapment, and ROS generation by GO, which collectively inhibit bacterial survival and limit the spread of infection [45].

### 2.5. Reproductive and Urinary Applications

The most common condition affecting the female reproductive tract is bacterial vaginosis, typically resulting from an imbalance characterized by the overgrowth of Gardnerella species and a reduction in protective Lactobacillus populations. Another common type of vaginitis is candidiasis, which is primarily brought on by *Candida albicans* (*C. albicans*). Nanomaterials have been investigated as intravaginal drug delivery carriers designed to enhance mucosal penetration, dispersion, and retention of therapeutic agents in the treatment of *C. albicans*-associated vaginitis owing to their favorable physicochemical properties [46]. Poly (lactic-co-glycolic acid) (PLGA), polyethylene glycol (PEG), and polyamidoamine (PAMAM) dendrimers have all demonstrated potential for constructing vaginal drug delivery systems. In contrast, GO-PEG nanoparticles have been reported to induce DNA damage and apoptosis in endothelial cells, leading to epithelial stress responses, programmed cell death, inflammatory cell recruitment, and exacerbated proinflammatory reactions in fungal vaginitis [47]. This finding highlights ongoing concerns regarding the biosafety of GO as an antimicrobial drug carrier. Additionally, there are still few studies examining the application of GO in treatments that target Gardnerella or Lactobacillus species, which is an area that needs more investigation.

Another significant clinical problem is urinary tract infections (UTIs), which are typically brought on by *S. aureus* or *E. coli*. Current treatment regimens typically last one to two weeks; however, recurrence rates remain high, given the excellent electrical conductivity of GO, the construction of electrochemical drug delivery systems capable of releasing antibiotics on demand is a promising therapeutic strategy for UTIs [48]. Nevertheless, there is currently a dearth of research on GO-based drug delivery systems that include frequently prescribed UTI treatments like trimethoprim-sulfamethoxazole or nitrofurantoin. Table 1 provides an overview of the representative examples.

### 2.6. Cross-System Comparison of the Antibacterial Applications of GO

Differences in the antibacterial efficacy of GO across infection sites depend not only on the pathogen involved but, more importantly, on whether sufficient local retention and effective pathogen contact can be achieved within a safe exposure range. Oral and cutaneous infections predominantly occur on tissue surfaces that are directly accessible for treatment. Oral applications focus primarily on inhibiting bacterial adhesion and biofilm formation; however, immobilized GO exhibits limited activity against surrounding planktonic bacteria. Its combination with antibiotics or remineralizing agents can broaden the antibacterial spectrum or simultaneously promote hard-tissue repair [22,23,24]. Given the rapid formation and continuous dynamic renewal of oral biofilms, GO-based materials should maintain stable interfacial antibacterial activity within an acceptable biosafety range while minimizing the risk of biofilm reformation. Wound dressings are required to remain in contact with the wound for prolonged periods. In addition to exerting direct antibacterial effects, GO often serves as a loading and dispersion platform for metallic nanoparticles, antibacterial polymers, or photoresponsive components, thereby integrating infection control with exudate management, angiogenesis, and wound healing [33,34]. The wound environment is particularly well suited to exploiting the stabilizing and dispersing functions of GO while maintaining a moist microenvironment and sustained antibacterial activity. In contrast, the membrane-disruptive and electron-transfer effects of GO in the gastrointestinal tract depend on the exposure of sheet edges and direct contact with bacterial cells. Although functionalization may enhance its inhibitory activity against planktonic bacteria, it does not necessarily produce a corresponding improvement in antibacterial activity against bacteria embedded within biofilms [29,30]. Gastric acid, mucus, and the complex gut microbiota may further compromise the antibacterial efficacy of GO by inducing sheet aggregation or forming a surface coating on the material. Given that gastrointestinal infections are commonly treated through oral administration, greater emphasis should be placed on the stability of GO-based systems within the gastrointestinal environment and their selective release at infection sites, rather than merely increasing the material concentration. In pulmonary infection models, pretreatment with GO has been shown to reduce pulmonary colonization and virulence of drug-resistant *K. pneumoniae* [18]. However, these findings do not establish the therapeutic efficacy of GO against established pulmonary infections, and post-infection aerosol administration models are still required to evaluate its pulmonary deposition, antibacterial efficacy, and tissue safety. Applications in the genitourinary tract are similarly constrained by clearance through body fluids and the sensitivity of mucosal tissues. Moreover, GO–PEG may induce cytotoxicity and inflammation in vaginal epithelial cells, suggesting that design strategies for this anatomical region should shift from broad-spectrum contact-mediated killing toward pathogen-selective binding and locally controlled antibacterial activity [47]. Overall, the rational design of GO-based antibacterial systems should prioritize site-specific local exposure, sustained pathogen contact, and durable antibacterial activity while minimizing adverse effects on host tissues and the resident microbiota.

## 3. Mechanisms Underlying the Antibacterial Activity of GO

**Figure 1:**
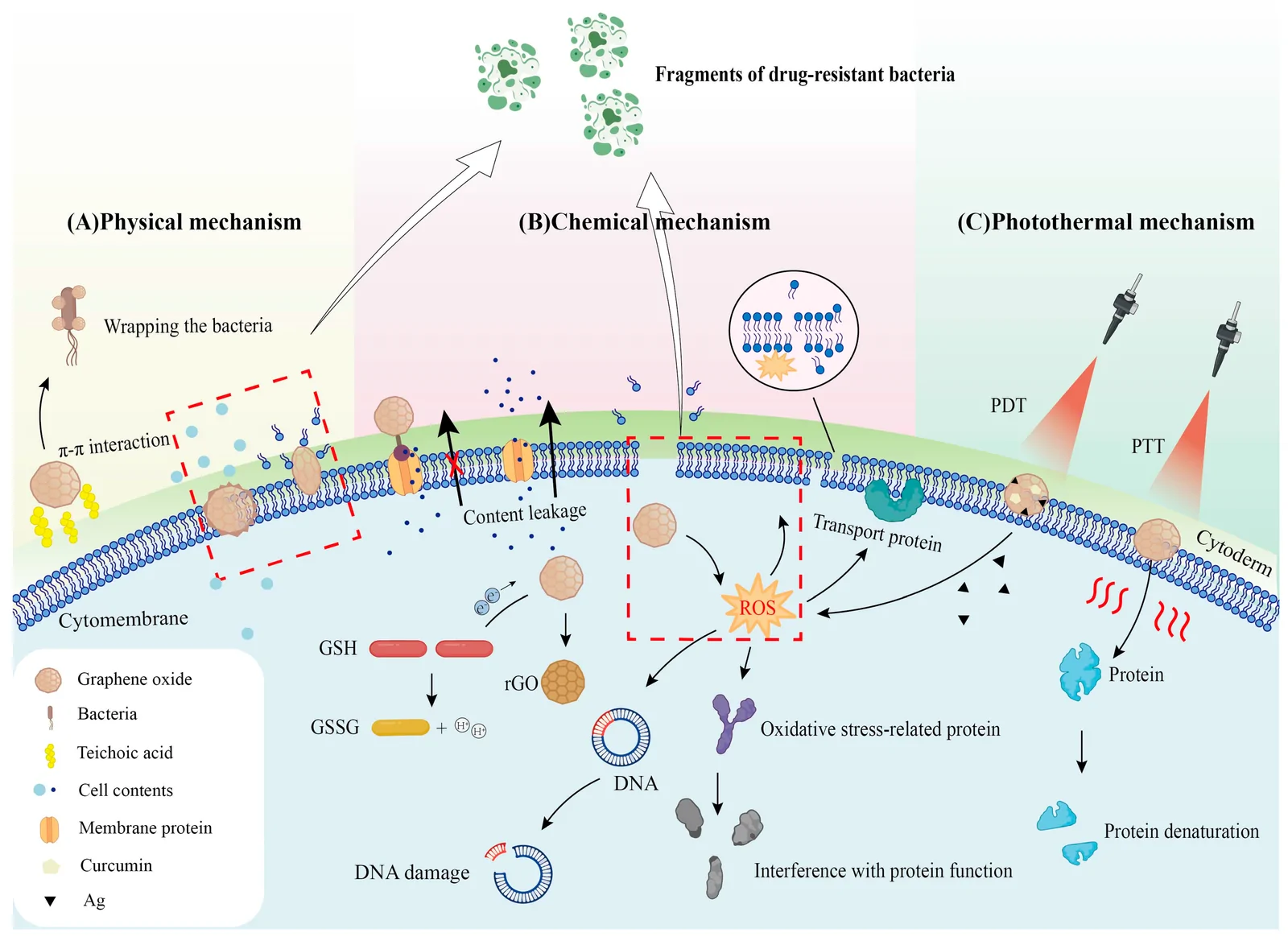
Mechanisms of GO Antibacterial Activity: (**A**). Physical Mechanisms of GO: (Left) GO facilitates bacterial encapsulation through π–π interactions with teichoic acids in the cell walls of Gram-positive bacteria; (Center) Sharp edges of GO cleave cell membranes, causing leakage of contents; (Right) The wrinkled surface of GO nanosheets physically damages cell membranes and inserts into them to extract phospholipid molecules. (**B**). Chemical Mechanisms of GO: (Left) Functional groups on GO surfaces bind to membrane proteins, preventing the leakage of contents.; (Right) GO can directly oxidize glutathione (GSH) via the functional groups on its surface. GO induces ROS production to damage cell membranes and interferes with the function of oxidative stress-related proteins and transporters. (**C**): Photo-responsive mechanisms of GO: (Left) PDT: GO loaded with curcumin promotes ROS generation and Ag^+^ release under photothermal effects; (Right) PTT: GO acts as a photosensitizer, generating photothermal energy under near-infrared irradiation, causing localized heating that denatures proteins. Additionally, GO disrupts bacterial membranes through its sharp edges and phospholipid-extraction activity, while inducing intracellular ROS generation—together conferring superior efficacy against drug-resistant bacteria.

Traditional antibiotics primarily exert their antibacterial effects by inhibiting the polymerization of peptidoglycan in the bacterial cell wall, compromising the integrity of the cytoplasmic membrane, interfering with nucleic acid and protein synthesis, and obstructing intracellular metabolic pathways, such as those involved in folate biosynthesis [49,50]. However, the extensive and often inappropriate use of antibiotics has led to the development of bacterial resistance against multiple conventional agents. The principal mechanisms of resistance include: (i) establishing physicochemical barriers to limit antibiotic entry into the cell; (ii) modifying drug targets; (iii) secreting specific hydrolytic enzymes that degrade antibiotics; (iv) activating efflux pumps to expel antibiotics; and (v) employing target protection strategies [51,52]. In contrast to the single-target mode of action characteristic of traditional antibiotics, GO exhibits multiple intrinsic antibacterial mechanisms as illustrated in Figure 1: (i) disrupting bacterial membranes through sharp-edge cutting [15]; (ii) capturing bacterial cells through a wrapping mechanism, thereby preventing them from accessing essential nutrients [53]; (iii) inducing a burst of intracellular ROS generation [54], leading to protein denaturation and DNA damage; (iv) acting as a photosensitizer, which under photo-responsive conditions can further amplify oxidative stress-induced damage through photothermal and photodynamic effects [55]. Importantly, this process operates independently of bacterial metabolic pathways, reducing the likelihood of resistance development.

### 3.1. Physical Mechanisms

The predominant antibacterial mechanism of pristine GO is based on their physical interactions with bacterial cells. The intermolecular interactions among bacterial membrane phospholipids (such as phosphatidylglycerol) are relatively weak, and their repulsive forces toward GO are low, which facilitates the approach and insertion of GO nanosheets with sharp edges into the phospholipid bilayer. This insertion process can be analogized to the mechanical cutting action of a “nanoknife,” which compromises membrane integrity, enhances membrane permeability, and ultimately induces membrane rupture and leakage of intracellular contents, culminating in bacterial cell death [56]. In addition, the wrinkled surfaces of the GO nanofilms further enhanced their antibacterial efficacy. The abundant depressions on their rough surfaces intensified the physical cutting effect on bacterial membranes, particularly when the dimensions of the depressions matched those of the microbial cells, with bactericidal efficiency being positively correlated with surface roughness [57]. At higher concentrations, abundant GO nanosheets may capture bacteria through a wrapping mechanism. Larger GO nanosheets can envelop entire bacterial cells, thereby isolating them from nutrients in the culture medium and indirectly inhibiting their proliferation [53,58]. Furthermore, owing to their large specific surface area and intrinsic hydrophobicity, GO nanosheets can effectively interact with and insert into the phospholipid bilayer of bacterial membranes. This interaction extracts significant amounts of phospholipids under suitable contact conditions, further compromising the membrane integrity and reducing bacterial viability [59,60]. Fourier transform infrared (FTIR) spectroscopy analysis revealed that after interaction between GO and Gram-positive bacteria, the absorption peak corresponding to the C=C bond exhibited a blue shift, supporting the involvement of π–π interactions between them. This interaction drives the specific and efficient adsorption of GO onto wall teichoic acids in the bacterial cell wall, subsequently inducing bacterial death by activating autolysins and intensifying peptidoglycan hydrolysis [61].

### 3.2. Chemical Mechanisms

Extensive research has demonstrated that GO can exert antibacterial effects through the induction of ROS, such as singlet oxygen (^1^O_2_) and superoxide anion radicals (O^2−^) [54,62,63]. These ROS oxidize unsaturated fatty acids, including phospholipids, within bacterial membranes, thereby compromising membrane integrity and permeability [64,65]. Simultaneously, ROS-mediated processes change the expression of membrane-associated and oxidative stress-related proteins and [66] cause cytoplasmic leakage and bacterial death through mechanisms like DNA damage [67]. In addition to ROS-dependent pathways, GO possesses the capability to directly oxidize intracellular reduced glutathione (GSH) in bacterial cells through its abundant oxygen-containing functional groups, such as carboxyl and epoxy groups. This oxidation process compromises GSH’s protective role against oxidative damage, leading to oxidative damage of other cellular components, including nucleic acids, proteins, and lipids. Notably, this mechanism does not depend on the production of ROS; rather, it diminishes the bacterial capacity to neutralize endogenous ROS through non-radical pathways [68]. Beyond directly participating in intracellular redox reactions, GO can also couple with bacterial extracellular electron transfer (BEET) to establish a bacteria-driven, self-powered antibacterial system. The GO substrate accepts extracellular electrons released during bacterial metabolism and transfers them to Cu^2+^, facilitating its reduction to Cu^+^ and subsequently generating hydroxyl radicals (·OH) through Fenton-like reactions, thereby disrupting bacterial biofilms and damaging essential biomolecules. Driven by electron signals from viable bacteria, this process enables responsive and sustained antibacterial activity, with no evident resistance observed after 129 consecutive generations of bacterial cultivation [69]. Furthermore, the surface functional groups of GO may form hydrogen bonds with bacterial secretory proteins, such as BamA, SurA, and SecY, interfering with membrane protein assembly and translocation processes [70]. Of particular note, a recent study in 2025 reported that the co-cultivation of GO with *Ralstonia solanacearum* for 48 h led to its reduction into rGO, which led to the induction of functional groups and oxidation states with high surface reactivity, and finally reduced the antibacterial properties of GO [53]. Similarly, *E. coli* has been observed to reduce GO, a phenomenon that may be linked to the hydrolytic enzymes and metabolic activity of surviving bacteria [71,72]. These findings suggest that bacterial resistance to GO via reduction processes represents an important challenge, underscoring the need for future studies to elucidate such resistance mechanisms in greater depth.

### 3.3. Photo-Responsive Mechanisms

Owing to its excellent responsiveness to near-infrared (NIR) light, GO is widely employed as a photothermal agent (PTA) in antibacterial photothermal therapy (PTT) [73]. Upon irradiation of the DS/PDA@GO-L dressing with 808 nm NIR light, GO efficiently converts optical energy into thermal energy. This process results in a rapid increase in local temperature exceeding 50 °C, which subsequently disrupts bacterial cell membrane structures, induces protein denaturation, membrane rupture, and leakage of cellular contents [74]. This approach offers distinct advantages over traditional antimicrobial strategies, as it reduces the likelihood of resistance development and effectively prevents biofilm formation, thereby holding great promise for clinical application [75]. In practice, NIR irradiation at wavelengths of 808, 795, or 875 nm is typically used to activate GO, which efficiently transforms light energy into heat. Within 10 min, local temperatures can exceed 50 °C, which is sufficient to denature bacterial proteins and eradicate pathogens, such as *E. coli* and *S. aureus*, while causing minimal damage to surrounding healthy tissues [76]. In addition to PTT, GO also serves as a versatile nanoplatform for photodynamic therapy (PDT). For instance, GO has been utilized to load and stabilize the photosensitizer curcumin while simultaneously incorporating AgNPs. This multifunctional system achieves enhanced antimicrobial efficacy through a combined ROS and Ag^+^ mediated mechanism, leading to fungal membrane disruption, suppression of virulence factor expression, and inhibition of biofilm formation, thereby providing potent activity against *C. albicans* [55]. In a similar manner, in GO/polydopamine–curcumin nanocomposites, GO stabilizes curcumin through π–π stacking interactions. Under white light irradiation in the range of 405–780 nm, curcumin is excited to generate large amounts of singlet oxygen (^1^O_2_), resulting in membrane rupture and content leakage in *S. aureus*, with a bactericidal rate reaching 99.99% [77]. These studies collectively highlight that GO primarily serves as a carrier and stabilizer in photodynamic systems, thereby significantly enhancing the antibacterial performance of photosensitizers.

Notably, no consensus has yet been reached regarding whether physical damage or oxidative stress constitutes the predominant antibacterial mechanism in the antibacterial activity of GO. We propose that these mechanisms do not act independently but instead undergo dynamic shifts depending on the material system and the surrounding environment. For pristine GO, membrane contact and disruption of membrane integrity generally represent the initial antibacterial events, with oxidative stress subsequently amplifying cellular damage [78]. In photoresponsive or metal-ion-containing composite systems, however, ROS-mediated oxidative damage may become the predominant bactericidal pathway [27,79]. Therefore, the mechanistic differences reported across studies are more likely attributable to variations in the physicochemical properties of GO and experimental conditions that alter its interfacial interactions with bacteria, rather than the existence of a universally dominant antibacterial mechanism.

### 3.4. Advantages of GO Against Drug-Resistant Bacteria

In comparison with conventional antibiotics, GO demonstrates distinct antibacterial properties against drug-resistant bacteria. GO effectively inhibits a range of clinically isolated multidrug-resistant (MDR) pathogens, including *E. coli*, *K. pneumoniae*, and *S. aureus*. This inhibition is achieved through mechanisms such as the “nano-knife” physical cutting effect of its sharp edges on bacterial cell membranes, the encapsulation and isolation effect of its large-sized sheets, and the induction of metabolic disturbances resulting from membrane integrity disruption and phospholipid extraction [31,80]. These distinctive physical killing mechanisms may reduce the likelihood of resistance arising through conventional target-specific mechanismsand even harder to develop resistance through genetic mutations. Moreover, GO can induce massive ROS generation and lipid peroxidation, causing extensive and non-selective oxidative damage to multiple biomolecules such as bacterial cell membranes, proteins, and DNA. As GO-induced oxidative damage simultaneously affects multiple cellular components, including membranes, proteins, and nucleic acids, its antibacterial activity is less likely to be fully circumvented by a single genetic alteration [11,81]. This multi-target attack strategy substantially diminishes the probability of bacterial resistance development and opens novel pathways for addressing drug-resistant infections. Furthermore, composite antibacterial agents, created by integrating GO with conventional antibacterial drugs or materials, can achieve a synergistic antibacterial effect that surpasses the sum of their individual effects. For instance, GO composites in conjunction with AgNPs exhibit more outstanding antibacterial efficacy against drug-resistant bacteria because of enhanced stability and dispersibility of AgNPs within the composites [82]. Zhang et al. [83] reported that prolonged exposure of *E. coli* to GO at 5 mg/L activated the Cpx envelope stress response, promoting biofilm formation, extracellular protease secretion, and cell membrane remodeling, thereby increasing bacterial tolerance to GO and acidic and oxidative environments, as well as enhancing adhesion to, invasion of, and intracellular survival within macrophages, ultimately increasing pathogenicity and pro-inflammatory potential. Studies have shown that at a GO concentration of 0.02 mg/L and under specific culture or environmental conditions, GO may promote bacterial aggregation and biofilm formation, thereby impairing bacteriophage infection and control of host bacteria, providing a protective microenvironment for bacterial survival, and increasing bacterial abundance and community diversity [84]. Therefore, despite its distinct advantages over conventional antibiotics, prolonged exposure to low doses of GO may still induce bacterial adaptive tolerance and alterations in microbial ecology. Future studies should further investigate bacterial adaptive responses, changes in pathogenicity, and biosafety during the long-term application of GO.

## 4. Comparison of the Antibacterial Performance of Pristine and Composite GO Systems and Optimization Strategies for GO-Based Composites

### 4.1. Comparison of the Antibacterial Performance of Pristine and Composite GO Systems

Functionalization can overcome several limitations of pristine GO, including its tendency to aggregate, limited stability, and variability in antibacterial efficacy. The enhanced antibacterial effects of composite systems may arise from GO itself, the incorporated active components, or both. In addition, GO can serve as a carrier or interfacial support to improve the loading, dispersion, stability, and release of active components, thereby enhancing overall antibacterial performance. For example, in GO–metal nanoparticle systems, the metal nanoparticles and their released metal ions generally exert potent direct bactericidal effects. In Ag/Cu/GO systems, Ag and Cu nanoparticles, together with their released metal ions, constitute the primary bactericidal components, whereas GO primarily serves as a supporting substrate for the nanoparticles; its surface oxygen-containing functional groups facilitate the in situ formation of metal nanoparticles and regulate metal-ion release [34]. In GO–antibiotic systems, antibiotics remain the principal active agents, while GO mainly enhances their therapeutic efficacy by increasing drug-loading capacity and prolonging drug release [85]. Similarly, in GO–photosensitizer systems, ROS generated upon light irradiation generally represent a major bactericidal factor. In the GO/PDA–Cur system, GO enhances the loading and stability of curcumin, while white-light irradiation induces ROS generation, resulting in bacterial killing [77]. Therefore, when evaluating GO-based composites, the key issue is not simply whether a composite outperforms pristine GO, but rather the specific functional role of GO within the system and which component predominantly accounts for its antibacterial activity.

### 4.2. Strategies for Optimizing GO-Based Composites

**Figure 2:**
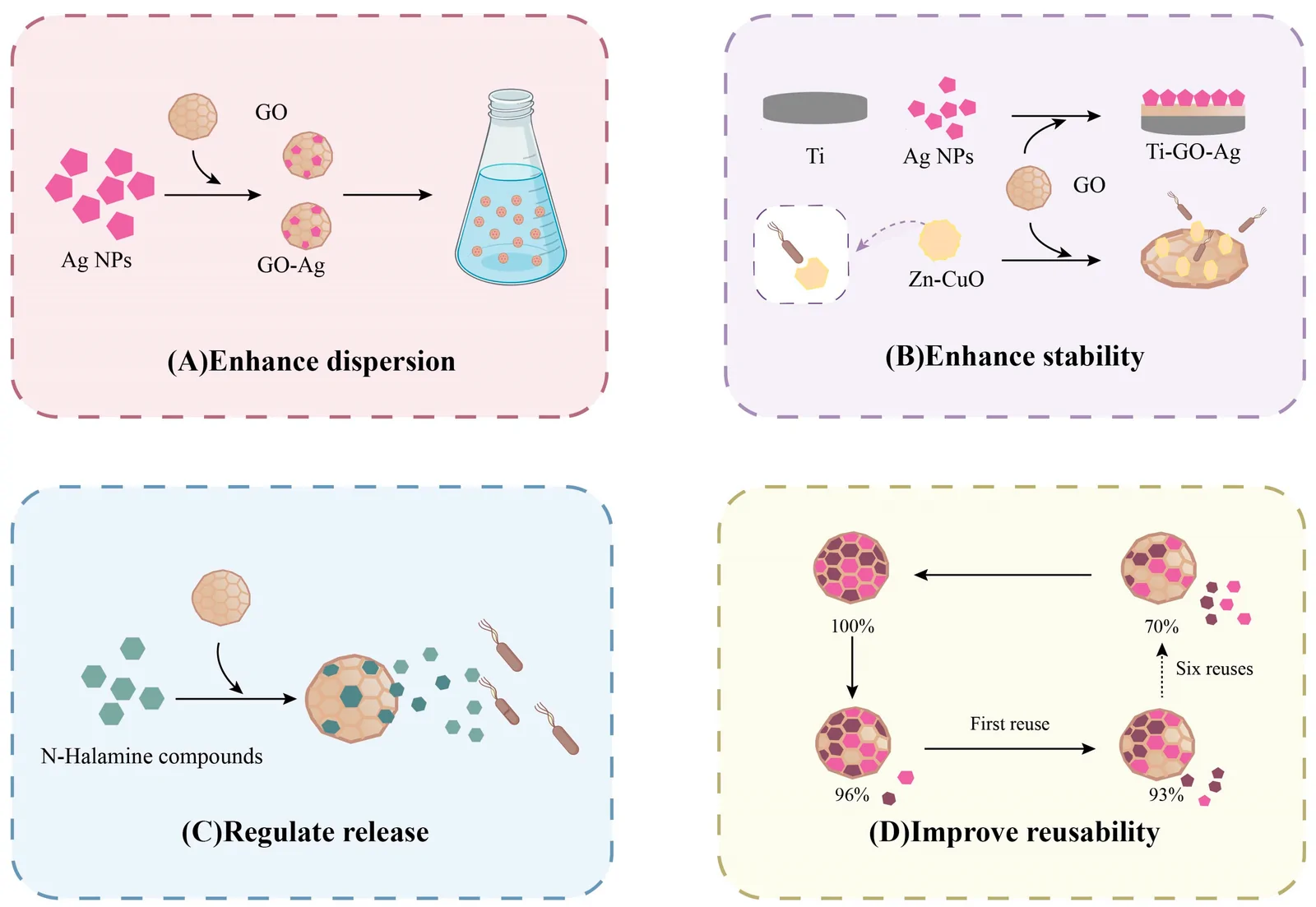
Optimization of GO composites: (**A**). Dispersibility of reinforcing materials: AgNPs can maintain good dispersibility in aqueous solution with the support of GO. (**B**). Stability of reinforcing materials: (Upper) GO acts as an interfacial binder, combining with the Ti matrix and AgNPs to enhance the stability of Ti-Ag nanocomposites; (Lower) Zn-CuO alone shows surface fragmentation defects, while the introduction of GO nanosheets enhances its stability. (**C**). Regulation of material release kinetics: GO is used as a carrier to load N-halamine compounds for sustained chlorine release, prolonging the sterilization time. (**D**). Enhancement of reusability of composites: MGO-Ag formed by loading AgNPs and Fe_3_O_4_ on the surface of GO maintains an inactivation rate of over 70% against *E. coli* after six uses.

In GO-based composite materials, it typically serves as a drug carrier to assist in exerting antibacterial effects [86,87,88]. As summarized in Figure 2, the optimization of GO-based composites focuses on several key aspects: dispersibility, stability, release rate of active agents, and reusability.

Good dispersibility is a prerequisite for antibacterial materials to cause physical damage. However, the spontaneous aggregation of nanomaterials in aqueous media reduces their active surface area and consequently diminishes antibacterial performance [44]. GO possesses excellent hydrophilicity and dispersibility in water, which makes it particularly effective for improving the solubility of composite materials [89]. For example, AgNPs supported by GO can maintain good dispersibility in aqueous solutions [90]. Compared with AgNPs alone, a GO-Ag nanocomposite (10 μg/mL) increased the killing rate of *E. coli* from 45% to 94% [91].

Stable interactions between antimicrobial agents and supporting matrices, as well as the stability of antimicrobial components in solution, are essential for maintaining the antibacterial efficacy of the composites. Metallic antimicrobial agents often exhibit weak binding to carrier substrates. GO can act as an interfacial binder, bridging both the matrix and antimicrobial components, thereby enhancing stability. For instance, in Ti-GO-Ag nanocomposites, GO mediates the robust integration of Ti and Ag nanostructures through electrodeposition and UV reduction methods [24]. Similarly, the spiky Zn-CuO nanostructure promoted stronger physical interactions at the bacteria-electrode interface. When Zn-CuO was present alone, its surface exhibited fragmented defects, whereas the introduction of GO nanosheets protected these fragile structures, resulting in superior stability [92]. In addition to enhancing interfacial interactions, surface functionalization can improve the colloidal stability of GO-based composites in physiologically relevant media. Bao et al. [93] modified GO–AgNPs with polyethylene glycol (PEG), which reduced material aggregation and sedimentation, allowing GO–AgNPs–PEG to remain stably dispersed for more than one week in physiological saline, phosphate-buffered saline (PBS), and various culture media, while exhibiting greater antibacterial activity than unmodified GO–AgNPs in these media.

GO can also regulate the release kinetics of antimicrobial components. By slowing down the release rate, GO prolongs antibacterial activity and reduces the potential toxicity associated with rapid release [94]. For instance, when loaded with N-halamine compounds, GO enables sustained chlorine release, thereby maintaining long-term bactericidal activity [95]. In another case, the oxygen-containing functional groups on GO provided abundant active sites for anchoring AgNPs, reducing the burst release of Ag^+^ [96]. Furthermore, the rough surface of GO increased the viscosity of the surrounding solution in Cu_x_O-GO composites, creating hydrodynamic resistance that hindered the diffusion of antimicrobial molecules [97].

The incorporation of recyclable components with GO can significantly improve the reusability of the composites. By combining magnetic nanoparticles with GO, magnetic separation of the composite material can be achieved, thereby facilitating repeated use. For example, magnetic nanocomposites composed of GO doped with Ag and Fe_3_O_4_ (MGO-Ag) retained over 70% bactericidal efficiency against *E. coli* even after 6 cycles of use. The embedded Fe_3_O_4_ nanoparticles not only conferred magnetic properties but also ensured facile recovery of the composite under an external magnetic field [98]. Several researchers have synthesized composites through the electrostatic assembly of GO with Fe_3_O_4_ nanoparticles modified with N-alkylated poly (4-vinylpyridine). When subjected to an external magnetic field, this material can be efficiently and completely separated from aqueous solutions within a span of 7 min. After undergoing 5 reuse cycles, the composite exhibits a slight reduction in its antibacterial activity against *E. coli*; however, it maintains an efficacy above 85%, thereby demonstrating commendable cycling stability and potential for reuse [99]. This study provides an important reference for the development of recyclable and environmentally friendly GO-based antibacterial materials.

## 5. Influence of Physicochemical Properties on Antimicrobial Efficacy of GO

The physicochemical properties of GO and its composites, including size, shape, adhesion degree, surface oxygen content, fabrication play a crucial role in determining their antibacterial performance.

### 5.1. Size

GO-based antibacterial materials exhibit size-dependent activity. Specifically, GO has both lateral and vertical structures. Larger lateral-sized GO nanosheets can more readily cover bacterial cells, thereby impeding their proliferation and leading to loss of viability. In contrast, smaller GO nanosheets tend to adhere only to the cell surface, making it difficult to effectively isolate cells from their surrounding environment [100,101]. Moreover, when the lateral size of GO decreases below 10 nm, as in the case of graphene quantum dots, the antibacterial activity drops sharply due to enhanced hydrophilicity and biocompatibility [102]. In contrast, small-sized GO exhibits stronger effects in terms of membrane cutting and DNA damage, owing to its larger specific surface area. For example, when the sheet area of GO decreases from 0.65 μm^2^ to 0.01 μm^2^, the antibacterial activity of GO-coated surfaces increases by nearly fourfold [62]. These seemingly contradictory findings suggest that size alone is not the sole determinant of GO-induced bacterial death [103]. Selecting GO within an appropriate particle size range enables better expression of its antibacterial properties.

### 5.2. Shape

The shape of the GO nanosheets is considered to directly influence their interactions with bacterial cell membranes [59,104]. Studies have demonstrated that GO nanosheets with sharp corners and protruding edges are more likely to penetrate bacterial membranes because this unique morphology reduces the energy barrier during the penetration process [105]. Of particular note is the contribution of surface curvature to bacterial killing. For instance, when comparing two types of graphene quantum dots (GQDs), C60-GQDs exhibited greater antibacterial efficacy against spherical bacteria than against rod-shaped species, owing to the higher compatibility between their Gaussian curvature and the curvature of spherical bacterial membranes, which facilitates binding and membrane disruption [106]. In addition, multilayered GO composites have been shown to display stronger antibacterial effects, which is likely attributable to the increased surface roughness and higher GO content of the films as the number of layers increases [107,108].

### 5.3. Adhesion Degree

The degree of adhesion between GO and bacterial cells also plays an important role in determining antibacterial performance. For example, researchers have developed composite cellulose fibers (CEL) doped with GO. *C. albicans* was found to adhere tightly to CEL, while *E. coli*, with its relatively thin cell membrane, could attach to both the surface and cross-sections of CEL. In contrast, *S. aureus*, characterized by its thick cell wall and spherical morphology, exhibited limited contact area with the material, rendering it less susceptible to GO-mediated damage and thereby more resistant to its antibacterial effects [109]. In another study, GO- and C60-derived graphene quantum dots (GQDs) were tested against multiple bacterial species. Interestingly, only the C60-GQDs demonstrated species-specific antibacterial activity against *S. aureus*, whereas *Bacillus subtilis*, *E. coli*, and *P. aeruginosa* remained unaffected. Observations via scanning electron microscopy (SEM) and atomic force microscopy (AFM) suggested that the antibacterial activity of GQDs may be linked to the compatibility between their Gaussian curvature and the surface curvature of the target bacteria [106]. Notably, the degree of adhesion between GO and bacteria is influenced by both the physicochemical properties of GO and the intrinsic characteristics of the bacteria; therefore, clarifying the upstream regulatory factors governing this interaction is more conducive to understanding the antibacterial mechanisms of GO.

### 5.4. Surface Oxygen Content

Research indicates that when the atomic percentage of surface oxygen atoms in GO reaches 30% of the total atomic composition, the high density of oxygen atoms compromises the structural integrity, imparting exceptional flexibility to the GO sheets. This “super-flexibility” facilitates extensive adherence of GO to bacterial surfaces in a parallel wrapping manner, leading to the disruption of membrane integrity through sustained mechanical stress and thereby achieving physical antibacterial effects. Conversely, when the surface oxygen content (SOC) is below 30%, the material’s rigidity increases significantly, altering its mode of action to the vertical insertion of sharp edges into the cell membrane. Therefore, SOC governs the transition of GO’s physical interaction mode from “flexible wrapping” to “rigid piercing.” [110]. Through the careful regulation of KMnO_4_ oxidant levels and reaction conditions, researchers synthesized a series of GO samples exhibiting progressively varying degrees of oxidation. Their findings indicated that samples characterized by high carboxyl content and low hydroxyl/epoxy content, attributed to flatter nanosheets, reduced thickness, and larger sp^2^domains, demonstrated superior adsorption capacity. This, combined with a “nanoknife” physical piercing effect, resulted in enhanced antibacterial activity [111]. Moreover, Tang et al. [112] reported that a concentration as low as 0.005 mol/L of Ca^2+^ can facilitate the restacking of GO nanosheets, augment the proportion of oxygen-containing functional groups, reduce electrostatic repulsion, and enhance van der Waals interactions. Additionally, Ca^2+^ form ionic bridges between GO and bacterial cells, promoting coprecipitation. This interaction results in nearly 100% bacterial removal efficiency at low bacterial suspension concentrations when GO is supplemented with 0.005 mol/L Ca^2+^. Moreover, the removal efficiency further improves with increasing Ca^2+^ concentrations. Future research that concurrently optimizes the SOC and the ionic environment of the solution could potentially develop an effective and controllable antibacterial strategy through physicochemical synergy.

### 5.5. Fabrication

Different synthesis methods mainly influence the antibacterial performance of GO by altering its degree of oxidation. The Hummers method and its modified versions are classic approaches for synthesizing GO [113]. By adjusting sulfuric acid dosage [114], reaction temperature or duration, and potassium permanganate amount [115], GO with varying oxidation degrees and defect densities can be prepared, and these defects and edge sites are believed to facilitate ROS generation, thereby enhancing antibacterial effects. Additionally, the Brodie method employs a fuming nitric acid and potassium chlorate system to oxidize graphite for the preparation of GO. Studies have shown that, compared with the Hummers method, GO prepared by the Brodie method exhibits significantly superior antibiofilm activity, mainly due to its stronger oxidation capability and higher surface oxidation degree [116]. However, this method involves the use of fuming nitric acid and potassium chlorate during synthesis, which may generate toxic gases and suffers from high cost and long reaction cycles, thereby limiting its large-scale production and practical application. Therefore, how to ensure homogeneity, cost-effectiveness, and environmental sustainability in large-scale GO production remains an important topic for future research.

**Figure 3:**
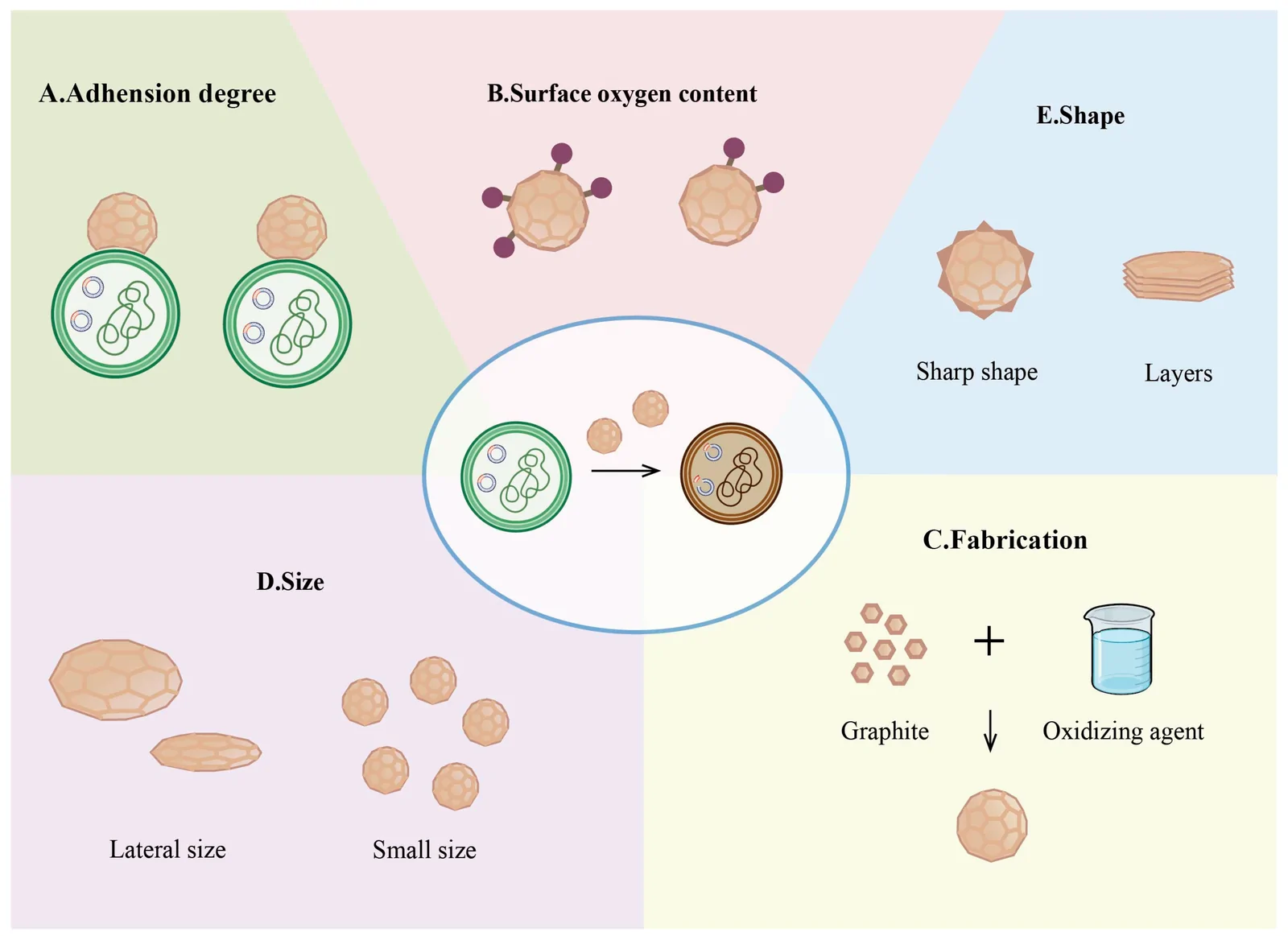
Physicochemical Properties affecting the antibacterial performance of GO and GO-based composites: (**A**). Adhesion degree: Intimate adhesion between GO and bacteria is a prerequisite for exerting its physical antibacterial action. (**B**). Surface Oxygen Content: The oxygen content on the GO surface dictates the mode of its physical antibacterial action. (**C**). Shape: Sharp shape and more layers enhance the antibacterial performance of GO. (**D**). Size: Both larger lateral size and smaller sizes can improve the antibacterial efficacy of GO. (**E**). Fabrication: The fabrication route governs GO’s structural stability and performance, thereby dictating its antibacterial efficacy.

The antibacterial activity of GO is modulated by various physicochemical parameters, including its size, morphology, surface charge, and fabrication process (summarized in Figure 3). It should be emphasized that the physicochemical parameters of GO, including sheet size, degree of oxidation, defect structure, and oxygen-containing functional groups, are not independent of one another but are interrelated during material preparation and collectively determine the mode of interfacial interaction between GO and bacteria. As a key factor linking structure and function, the degree of oxidation regulates the distribution of oxygen-containing functional groups, the integrity of the sp^2^ carbon framework, and the electronic properties of the surface, thereby influencing its hydrophilicity, dispersion behavior, and interactions with bacterial cells [117]. Meanwhile, variations in sheet size not only alter the contact area between GO and bacteria but also regulate edge density and defect exposure, making smaller GO sheets more likely to exert membrane-cutting effects, whereas larger sheets tend to act through bacterial encapsulation and nutrient deprivation [118]. By modulating the surface oxidation state of GO, Barrios et al. found that variations in the degree of oxidation not only affected its chemical reactivity but also reshaped its mode of interaction with bacteria. Highly oxidized GO primarily induced oxidative damage by promoting intracellular ROS generation and GSH depletion, whereas the reduction in oxygen-containing functional groups gradually shifted its antibacterial action toward a physical mode characterized by bacterial adsorption, capture, and growth inhibition [117]. This study demonstrated that the degree of oxidation is not a single determinant of the antibacterial performance of GO but instead influences its overall antibacterial effects through the coordinated effects of surface chemistry, electronic structure, and interfacial interactions.

## 6. Other Factors Influencing the Antimicrobial Efficacy of GO

### 6.1. Variations in Bacterial Species

The responsiveness of different bacterial species to GO also varied considerably. Several studies have reported that GO exhibits stronger antibacterial activity against Gram-positive bacteria than Gram-negative species. The outer membrane of Gram-negative bacteria serves as a protective barrier, restricting GO primarily to physical cutting mechanisms. In contrast, the more porous cell wall structure of Gram-positive bacteria, together with their thick peptidoglycan layer and clustered growth pattern, increases the effective contact area with GO, making them more susceptible to wrapping and subsequent growth inhibition [116,119]. Wang [61] et al. found that GO can efficiently adsorb teichoic acids unique to the cell walls of Gram-positive bacteria through π–π interactions. The absence of teichoic acids leads to dysregulated autolysin activity, excessive hydrolysis of peptidoglycan, and ultimately collapse of the cell wall structure, resulting in bacterial death. Furthermore, some studies suggest that Gram-positive bacteria can exploit surface peptidoglycans as chelating agents to strengthen interactions with nanomaterials, thereby enhancing contact-mediated bactericidal effects [120].

Some bacteria display unique resistance to GO-mediated antibacterial effects. For instance, *S. mutans* exhibits stronger antioxidant capacity than *Enterobacter cloacae*; after 12 h of treatment, its ROS levels remain significantly lower, thereby preserving membrane integrity [121]. In the case of GO-based materials that utilize photothermal effects, differences in bacterial heat tolerance also contribute to variable antibacterial outcomes. *S. aureus* demonstrates slightly greater heat resistance than *E. coli*, resulting in shorter eradication times for *E. coli* when treated with GO hydrogels under photothermal conditions [69]. Conversely, one study developed a system in which photothermal stimulation triggered the release of hyaluronidase by *S. aureus*. The released enzyme facilitated the liberation of AgNPs within the system, thereby enhancing antibacterial efficiency. As a result, this platform exhibited superior antibacterial activity against *S. aureus* compared to *E. coli* [122].

Current studies of the antibiofilm activity of GO are largely limited to single- and dual-species in vitro models. Fallatah et al. [123] found that GO accumulated on the surface of mature *Pseudomonas putida* biofilms and significantly reduced the number of viable bacteria within the biofilms through sustained contact with sessile cells and disruption of cell membrane integrity. In a dual-species chronic wound biofilm model comprising *S. aureus* and *P. aeruginosa*, Di Lodovico et al. [124] found that GO interfered with bacterial aggregation and biofilm stability by enveloping bacterial cells and altering the mobility of the fibrin network. Iosub et al. [125] developed an HAp/GO/ceftazidime composite coating that significantly inhibited the early colonization and biofilm development of *S. aureus* and *E. coli* on titanium surfaces. Studies on multispecies biofilms remain limited, likely owing not only to the difficulty of model establishment but also to differences among microorganisms in growth rates and nutritional and oxygen requirements, making it difficult to maintain a stable community composition under uniform culture conditions. In addition, synergistic or competitive interactions may occur among different microbial species, and the resulting biofilm structure is readily influenced by species proportions, inoculation sequence, and culture conditions, thereby limiting experimental reproducibility.

Compared with bacteria, fungi exhibit more complex cellular structures and growth forms, with a cell wall composed of β-glucans, chitin, mannoproteins, and other components surrounding the cell membrane, and may occur as yeast cells, spores, or hyphae [126]. Current research on GO and its composites against clinically relevant fungi has focused primarily on *Candida* species. In curcumin-containing composite hydrogels, GO contributes to the formation of the drug-loading and supporting matrix and may also exert direct antifungal effects through its exposed sheet edges [127]. In Ag/GO and Ag/rGO systems, GO or rGO is primarily used to immobilize and disperse AgNPs, thereby enhancing the interactions between the composite materials and fungal cells [128].

### 6.2. Environmental Factors

Bacterial growth conditions, including pH and temperature, can significantly influence the antibacterial activity of GO. Specifically, environmental pH alters the chemical stability of GO composites and the redox kinetics of metallic nanoparticles, thereby modifying their antibacterial efficacy [98,129]. For instance, in GO-AgNPs systems, the release of Ag^+^ originates from the oxidation of AgNPs within the composite. An increased proton concentration promotes the forward direction of the oxidation reaction, producing more Ag^+^; thus, lower pH values result in higher levels of Ag^+^ generated in the solution [129]. Temperature can alter the structure of GO itself. Bu [130] et al. observed that when the ambient temperature rises to 300–1000 K, hydroxyl and epoxy groups on the GO surface preferentially dissociate, while carboxyl groups begin to decompose at around 800 K, releasing H_2_ and CO_2_. One study compared the antibacterial effects of MGO-Ag against *E. coli* and *S. aureus* at different temperatures and found that its efficacy increased with increasing temperature. When the temperature was elevated to 37 °C and 45 °C, both bacterial strains exhibited nearly 100% loss of viability. This may be attributed to the fact that higher temperatures not only enhance the susceptibility of bacterial membranes to nanoparticle-induced physical damage, but also accelerate silver ion release and oxidative stress, thereby disrupting the structure and function of bacterial cells [98].

It is worth noting that environmental factors such as pH and temperature also exert a substantial influence on bacterial viability. Therefore, when evaluating the antibacterial performance of GO-based composites, it is essential to consider the potential confounding effects of environmental conditions on bacterial activity.

### 6.3. Mixing Ratio

Within a certain range, the antibacterial performance of the composites was positively correlated with the mixing ratio of GO. For example, in poly (propylene fumarate)-based nanocomposites, the sample with 3.0 wt% GO loading exhibited the strongest antibacterial activity, likely owing to the increased interfacial contact area between the bacteria and nanomaterials as the GO content increased [131]. In polyhydroxyalkanoate (PHA)/GO composite films, an incorporation of GO at 2 wt% achieves an antibacterial efficacy approaching 100%, which is considered the optimal concentration. Conversely, increasing the GO content to 5 wt% results in a marked decline in antibacterial performance. This reduction is primarily attributed to the tendency of GO nanosheets to restack and aggregate at higher concentrations, leading to a diminished effective edge density and accessible surface area. Consequently, this aggregation impairs physical interactions with bacterial cells, reduces membrane-disruptive effects, and decreases the efficiency of ROS generation, thereby contributing to the observed decline in antibacterial activity [132]. Similarly, in chitosan chloride-graphene oxide (CSCl@GO) systems, antibacterial activity initially improved with increasing GO content but declined once the mass fraction of GO exceeded 0.6%. This effect can be attributed to the fact that CSCl@GO carries a positive charge, enabling electrostatic adsorption onto the negatively charged bacterial surface. However, excessive GO promotes amidation reactions with CSCl, consuming a substantial number of amino groups and thereby reducing the positive charge density, ultimately leading to diminished antibacterial efficacy [133]. Due to differences in antibacterial mechanisms, the effect of GO content on the antibacterial performance of composite materials varies significantly. Similar to the influence of particle size, optimizing the mixing ratio can effectively enhance their antibacterial efficacy.

### 6.4. Exposure Time

**Figure 4:**
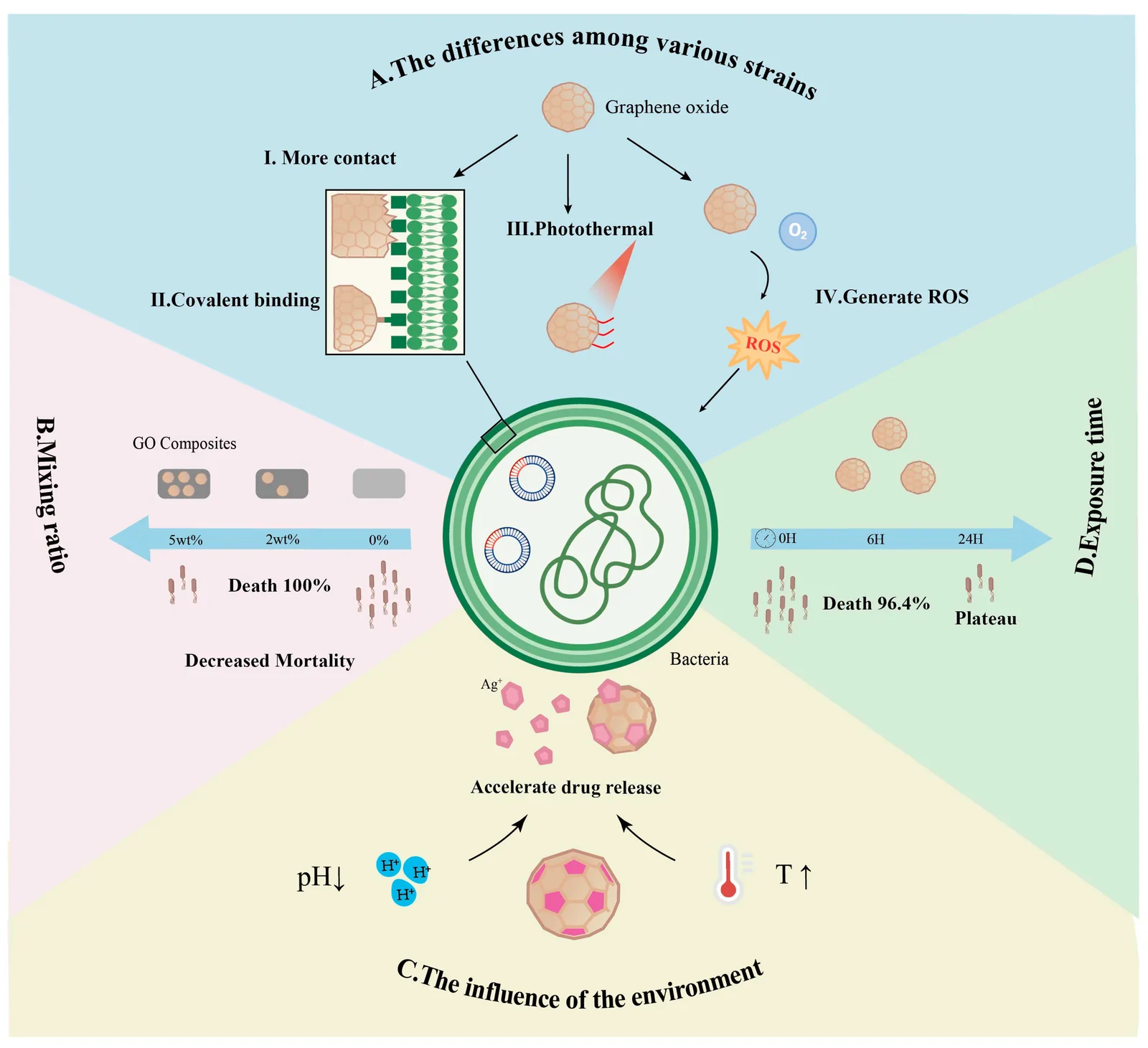
Factors affecting the antibacterial performance of GO and GO-based composites: (**A**). Microbial species: (I) GO increases membrane contact area to kill Gram-positive bacteria; (II) Gram-positive cell-surface peptidoglycan can chelate and thereby augment GO contact-killing; (III) GO photothermal effects kill *Escherichia coli*; (IV) GO-generated ROS can kill *Enterobacter cloacae*. (**B**). Mixing ratio: At 2 wt% GO loading, bacterial inhibition approaches 100% (optimal efficacy), whereas at 5 wt%, the antibacterial performance decreases significantly. (**C**). Environmental factors. Lower pH and higher temperature can promote Ag^+^ release from GO composites, thereby increasing antibacterial efficacy. (**D**). Exposure time: GO exhibits a kill rate of 96.4% within 6 h, which plateaus upon further exposure up to 24 h.

The duration of the interaction between GO-based antimicrobial composites and bacteria also plays an important role in determining the antibacterial performance. In general, longer exposure times result in higher bactericidal rates. For instance, the antibacterial efficacy of GO-MoS_2_ against *E. coli* increased with time, achieving a killing rate of 96.4% after 6 h [134]. The antibacterial efficacy of MGO-Ag against *E. coli* can rapidly increase to 90% within 30 min and reach a stable level of 100% during the subsequent 30–60 min [98]. However, some studies have indicated that prolonged exposure may lead to a plateau phase, during which further improvements in antibacterial efficacy occur only gradually. Identifying the optimal exposure duration is essential when considering the practical application of GO-based antimicrobial strategies. Standardizing the timing of administration can lead to effective antibacterial outcomes while minimizing excessive drug use, thereby reducing waste and the risk of inducing antimicrobial resistance. To better visualize the factors affecting the antibacterial performance of GO, we have summarized them in Figure 4.

## 7. Biosafety of Graphene Oxide

Although GO shows great application potential in the antibacterial field, its potential biosafety risks cannot be ignored. A large number of in vitro and in vivo studies suggest that the biological toxicity mechanisms of GO may be closely related to the oxidative stress it induces [135,136,137]. Animal models have also confirmed that intraperitoneal injection of 5 mg/kg body weight of GO in mice can induce toxic responses such as liver edema and inflammation [138]. At higher doses (10 mg/kg body weight), GO exposure for only 24 h can cause liver injury and acute nephrotoxicity in rats [139]. These findings suggest that GO may induce damage to organs such as the liver and kidneys within a relatively short period after entering the body, and its safety assessment should therefore fully consider material properties, administered dose, and exposure duration.

After entering the bloodstream, GO can also interact directly with erythrocytes and plasma proteins. Studies have shown that GO not only disrupts erythrocyte membrane integrity and induces hemolysis but also alters the conformations of albumin and fibrinogen, thereby activating the complement system and interfering with coagulation, suggesting a potential disturbance of blood homeostasis [140]. GO can subsequently be internalized by macrophages and activate TLR4-dependent inflammatory signaling, inducing necroptosis accompanied by ROS accumulation, cytoskeletal remodeling, and impaired phagocytic activity, thereby weakening innate immune function and amplifying inflammatory responses [141]. Therefore, the effects of GO on hemocompatibility and immune homeostasis also warrant particular attention.

Regarding in vivo metabolism and clearance, small and thin GO nanosheets are primarily eliminated through the kidneys and urinary tract, whereas the fraction not promptly cleared can be taken up by macrophages in the splenic marginal zone and undergo gradual intracellular degradation over an observation period of up to nine months. No evident pathological damage to the spleen was observed during this period, suggesting that long-term retention of GO does not necessarily result in persistent toxicity [142]. However, repeated intraperitoneal administration may cause dose-dependent abnormalities in liver function indicators, oxidative damage, and tissue inflammation [143], whereas prolonged respiratory exposure may activate caspase-1-, p38 MAPK-, and TGF-β1-related pathways, inducing pulmonary fibrosis and damage to the liver and spleen [144]. These findings indicate that the long-term safety of GO depends on multiple factors, including sheet size, exposure dose, route of administration, and exposure duration, and therefore cannot be determined from a single exposure model.

In addition to the intrinsic physicochemical properties of GO, the protein corona formed after its entry into biological environments is an important determinant of its in vivo behavior and biocompatibility. This protein corona can significantly alter its distribution profile, immune recognition, and circulation time [145]. Changes in the composition of the protein corona, particularly the reduced formation of a stable hard protein corona, have been demonstrated to be associated with more severe acute lung and liver injury in vivo [146]. Moreover, protein coronas formed on differently modified surfaces may lead to variations in recognition by the mononuclear phagocyte system, platelet depletion, thrombosis, and inflammatory responses, while PAA-functionalized GO exhibits favorable in vivo biocompatibility because of the formation of an opsonin-poor protein corona [147]. However, the protein corona may also mask the active surface of GO and weaken its antibacterial activity. A bovine serum albumin (BSA) corona reduced the photothermal bactericidal efficacy of GO against MRSA from 94.3% to 62.3% [148]. Further studies have shown that noncovalent adsorption onto the basal plane of GO can similarly attenuate its intrinsic antibacterial activity, with the addition of only 10% Luri-Bertani (LB) medium to physiological saline almost completely abolishing its bactericidal activity [149]. Therefore, although the protein corona may reduce host toxicity, it can also compromise the antibacterial activity of GO. Its dynamic evolution further increases the complexity of GO behavior in vivo, limiting the ability of in vitro assessments to accurately predict therapeutic efficacy and biosafety, and thereby representing an important barrier to clinical translation.

## 8. Limitations and Future Perspectives

Studies indicate that GO possesses broad-spectrum antibacterial activity against a wide range of Gram-positive and Gram-negative bacteria [61]. The oral cavity, respiratory tract, and gastrointestinal tract all exhibit complex microecological structures in which multiple microbial communities coexist [150,151]. In such complex microecological environments, the broad-spectrum antibacterial properties of GO may non-selectively eliminate beneficial microbiota, thereby disrupting microecological balance [152]. The ability of GO and its composites to selectively target pathogenic species within these complex settings, while maintaining specificity, remains an open question. Future research should employ advanced animal models or develop in vitro simulation systems, such as those replicating the oral microecological environment, to assess whether GO-based materials can achieve pathogen-specific antibacterial effects amidst microbial coexistence.

While exerting antibacterial effects, the potential regulatory impact of GO on the host immune system warrants close attention. Some studies have found that GO may interfere with immune balance by affecting the differentiation of host immune cells. PEG-modified GO exhibits significant immunomodulatory capacity in pro-inflammatory environments by selectively reducing the proportion of Th2 helper T-cell subsets while increasing Th9 subsets, and this immune shift may potentially affect host immune homeostasis [153]. Therefore, this regulatory mechanism should be considered an important factor in evaluating the safety of GO antibacterial applications to prevent side effects or diseases induced by immune imbalance. Furthermore, GO-based nanosystems do not rely solely on the independent action of GO but also influence the host immune system through the synergistic effects of functional modification and drug loading. For example, Pi [154] et al. developed a multifunctional drug-loaded nanosystem with GO as the core framework. By loading carbamazepine, a core autophagy inducer, this system significantly promotes autophagy and apoptosis in macrophages infected with *Mycobacterium tuberculosis* and drives their polarization toward the pro-inflammatory M1 phenotype. This synergistic regulatory strategy provides a new direction for enhancing immune-mediated clearance of infections using GO nanomaterials, but its long-term impact on host immune homeostasis still requires further investigation.

GO still faces multiple obstacles on its path toward large-scale clinical application. Our previous studies have shown that batch-to-batch variation in GO production, stability issues, and storage conditions all hinder its clinical translation [155]. Although current characterization techniques such as Raman spectroscopy, XPS, FTIR, and XRD can elucidate material properties, results from different laboratories are not directly comparable due to batch variations arising from synthesis methods, highlighting the lack of standardized data interpretation and evaluation systems [156]. Previous studies have revealed substantial variability in the quality of commercially available GO products. A systematic evaluation of 34 commercial GO products found that only four samples met the predefined acceptability criteria and were broadly consistent with their label information; approximately half contained metal residues exceeding 5000 ppm, only about six could form continuous, uniform, and stable films, and their sheet resistance ranged from 0.85 to 157.12 MΩ/sq [157]. These findings indicate that differences in residual impurities and the degree of oxidation may directly affect material performance. Such variability reflects not only differences in quality control among suppliers but also the high sensitivity of GO synthesis to processing parameters. Reaction temperature, reaction time, and oxidant ratio can alter the oxidation state of carbon and product yield, while GO with a higher degree of oxidation exhibits a more negative zeta potential, a smaller hydrodynamic size, and a higher critical coagulation concentration. Sonication time and the initial suspension concentration further influence the exfoliation yield and the composition of the final product [158]. Given that these parameters are closely associated with antibacterial activity, future studies should not only standardize preparation conditions but also establish quantitative relationships between key structural parameters and antibacterial performance.

The evaluation of the antibacterial performance of GO likewise lacks standardized criteria [159]. Although numerous studies have reported high bactericidal efficacy for GO [18,39], these findings have generally been obtained using heterogeneous experimental systems. Influencing factors include the bacterial inoculum, GO concentration, exposure duration, culture conditions, and detection methods, and variations in these parameters limit the comparability of antibacterial efficacy reported across studies. Therefore, a standardized antibacterial evaluation protocol should be established by defining the bacterial inoculum concentration, the tested range of GO concentrations, the exposure duration, and uniform outcome measures. In addition, when reporting bactericidal rates, researchers should provide detailed parameters of GO and related materials, including lateral size, thickness, degree of oxidation, C/O ratio, and zeta potential, to ensure that comparisons among studies are based on materials with similar characteristics. Formulation stability is another important factor limiting the further application of GO. GO is neither completely stable nor chemically inert, and its structural and chemical properties may change over time. Studies have shown that the intrinsic stability of GO at 298 K can be maintained for only approximately five days, after which changes may occur in the composition and structure of its oxygen-containing functional groups [160]. During prolonged exposure to oxygen-containing aqueous systems, GO may also undergo carbon-framework cleavage through pathways involving superoxide radicals (O_2_•^−^), hydrogen peroxide (H_2_O_2_), and hydroxyl radicals (·OH), resulting in the formation of small molecular fragments [161]. Therefore, GO should be stored under dry conditions, under vacuum, or in an inert atmosphere to minimize its contact with moisture, oxygen, and reactive substances. For GO dispersions, storage duration should be minimized, and particle size, oxygen-containing functional groups, and dispersion stability should also be assessed before use to reduce the effects of material aging on antibacterial performance and experimental reproducibility.

In recent years, emerging materials such as MXenes and black phosphorus have also been widely investigated for antibacterial and antibiofilm applications. Both MXenes and GO are two-dimensional sheet-like materials that can exert antibacterial effects through sheet–cell contact, membrane disruption, and oxidative stress; compared with GO, MXenes generally exhibit superior electrical conductivity and photothermal properties [162]. Rasool et al. [163] compared the antibacterial effects of TiC2T_x_ MXene and GO against *E. coli* and *Bacillus subtilis* under identical concentration and exposure conditions. Both materials exhibited concentration-dependent antibacterial activity; however, after treatment at 200 μg/mL for 4 h, TiC2T_x_ achieved inactivation rates exceeding 98% against both bacterial species, compared with approximately 90% for GO. However, subsequent claims that MXenes exhibit superior antibacterial performance to GO have largely been based on this study and lack independent head-to-head validation; therefore, MXenes cannot yet be considered universally superior to GO across different bacterial species and application conditions. Black phosphorus is a layered two-dimensional semiconductor with strong photoresponsiveness and biodegradability. Previous studies have directly compared black phosphorus nanosheets with GO and reported that black phosphorus exhibited greater antibacterial activity under specific experimental conditions [164]. Overall, direct comparisons of the antibacterial performance of GO with that of other emerging materials remain limited, and further evidence is required to substantiate these findings.

Current research predominantly focuses on short-term exposure models within 24 h [139,165], while systematic data regarding the long-term fate and chronic accumulation effects of GO in vivo remain insufficient. This limitation substantially restricts the comprehensiveness of its risk assessment. To facilitate the transition of GO from laboratory research to clinical application, it is imperative to achieve standardization of production processes, elucidate the underlying independent mechanisms, and establish regulated translational pathways.

## 9. Summary

This review systematically synthesizes the advancements in research concerning GO and its composite materials within the antibacterial domain. It offers a comprehensive analysis of the various factors affecting the antibacterial efficacy of GO, including its physicochemical properties, such as particle size, surface charge, and oxygen content, as well as processing methodologies, variations in bacterial species, environmental conditions, and duration of exposure. The article highlights that, in comparison to conventional antibiotics, GO presents distinct advantages in addressing drug-resistant bacterial infections. These advantages arise from its multifaceted mechanisms of action, including physical disruption and the induction of extensive oxidative stress, which are less prone to fostering bacterial resistance, alongside its broad-spectrum antibacterial activity. Furthermore, the development of composite antibacterial materials leveraging the unique physicochemical characteristics of GO can enhance the dispersibility, stability, sustained release, and recyclability of traditional materials. By integrating multidimensional mechanisms including “physical damage–oxidative stress–photo-responsive enhancement”, GO-based composite materials provide promising new strategies for the prevention and control of drug-resistant bacterial infections. Future research should focus on optimizing in vivo models, meticulously evaluating the potential immunological risks associated with GO, and enhancing the uniformity of large-scale production processes to facilitate more effective clinical translation.

## Figures and Tables

**Table 1:** Antibacterial Behavior of GO and Its Composite Materials.

Applications	Material Category	Synthesis of GO	Size	Raman Characterization	Zetapotential	Bacterial Species	Mechanism of Action	Antibacterial Property (Survivalrate)	In Vivo Validation	Refs.
Oral field (Prevention of dental caries)	GO nanosheets	Modified Hummers’ method	Size: 200–400 nm	I_D/I_G = 1.03	/	*S. mutans* (*Streptococcus mutans*)	Inhibit *E. coli* growth: (1) insert/Cut through the cell membranes. (2) extract large amounts of phospholipids from the membranes.	20 μg/mL: 44.2 ± 2.2% 40 μg/mL: 32.2 ± 7.9% 80 μg/mL: 16.3 ± 2.1%	/	[15]
	GO	Pyrolytic carbonization of citric acid	/	/	/	*S. mutans* (*Streptococcus mutans*)	Inhibit biofilm formation: (1) sharp edges damage the cell membrane. (2) generate oxidative stress.	0.25 mg/mL: 20% 1.25 mg/mL: 0%	/	[23]
Oral field (Prevention of periodontal infections)	GO (Drug carrier)	Commercially available GO	/	D band ≈ 1350 cm^−1^ G band ≈ 1580 cm^−1^	/	*S. mutans* (*Streptococcus mutans*)*; E. coli (Escherichia coli*)	Inhibit *S. mutans*, *E. coli* growth: (1) contact-killing. (2) release minocycline.	/	/	[24]
Oral field (root canal treatment; dental pulp regeneration)	CPC-chitosan-GO paste	Commercially available GO	/	/	/	*E. Faecalis* (*Enterococcus faecalis*)	Inhibit *E. faecalis* growth: (1) physical sharp edge as “nano-knife” puncturing. (2) trapping the bacteria.	50 μg/mL: 55.8 ± 4.8%	/	[27]
Gastrointestinal tract	GO nanosheets	Commercially available GO	Thickness: 0.8 nm	/	/	*E. coli* (*Escherichia coli*)	Inhibit *E. coli* growth: (1) physical membrane disruption. (2) electron-transfer oxidation.	/	/	[29]
	GO nanoribbons	Modified Hummers’ method	Width: 200 nm	D band ≈ 1350 cm^−1^ G band ≈ 1570 cm^−1^	/	*E. coli* (*Escherichia coli*)	Inhibit *E. coli* growth: (1) removed the essential nutrients via adsorption. (2) ROS generation.	/	/	[16]
Gastrointestinal tract	GO nanosheets	Commercially available GO	Thickness: 1.02 nm	/	−34.13 mV	*MRSA* (*Methicillin-resistant Staphylococcus aureus*)	Inhibit MRSA growth: (1) wrapping adsorption between GO and MRSA (2) physical cutting	5 μg/mL: 97.28%, 80.79%, 73.98%(30, 90, and 180 min) 10 μg/mL: 87.44%, 62.48%, 57.19% (30, 90, and180 min) 20 μg/mL: 53.86%, 42.21%, 35.70% (30, 90, and 180 min)	/	[31]
Skin(wound dressing)	PVA/GO/Av (Hydrogel)	Commercially available GO	/	PVA/GO: ID/IG = 0.62 PVA/GO/Av: ID/IG = 0.77	/	*S. mutans* (*Streptococcus mutans*)	Inhibit *S. mutans* growth: (1) cut the cell membrane mechanically (2) ROS generation (3) bacterial membrane entrapment	0.47 ± 0.045%	/	[33]
Skin (Promote wound healing)	Ag/Cu/GO nanocomposites	Commercially available GO	Nanoparticles on GO surface: 7.25 nm	/	/	*P. aeruginosa* (*Pseudomonas aeruginosa*)	Inhibit *P. aeruginosa* growth: (1) physical damage (2) release metal ions	50 μg/mL: 0.6 ± 0.06%	*P. aeruginosa*-infected mouse skin wound model	[35]
Skin (Promote wound healing)	Ag/GO-GelMA (hydrogels)	Commercially available GO	/	D band ≈ 1338 cm^−1^ G band ≈ 1597 cm^−1^	GO: −31.1 mV; Ag: −1.1 mV; Ag/GO: −21.6 mV	*S. aureus* (*Staphylococcus aureus*)*; E. coli* (*Escherichia coli*)	Killing *E. coli* and *S. aureus*: (1) physical damage (2) ROS generation (3) the “photothermal synergy” mechanism with Ag	*S. aureus*: 0.8% *E. coli:* 0.4%	Infected wound model in Sprague–Dawley rats	[39]
Skin (Accelerated healing process)	C/H/GO/Cu dressings (GO/Cu-decorated chitosan/hyaluronic acid dressings)	Modified Hummers’ method	/	/	/	*MSSA* (*methicillin-susceptible Staphylococcus aureus*)*;* *MSSE* (*methi cillin-susceptible Staphylococcus epidermidis*)	Inhibit bacterial adherence and subsequent biofilm formation: (1) physical damage (2) oxida tive stress	MSSA, 16 μg/mL: 0% MSSE, 8 μg/mL: 0%	Infected full-thickness skin defect model in BALB/c mice	[34]
Respiratory tract	GO nanosheets	Commercially available GO	/	/	−45 mV	*Kp* (*Klebsiella pneumoniae*) *E. coli* (*Escherichia coli*) *and P. aeruginosa* (*Pseudomonas aeruginosa*)	Inhibit bacteria growth: (1) cellular trauma with sharp edges. (2) wrapping or trapping the bacteria. (3) ROS generation	Kp, 62.5, 125, 250 and 500 μg/mL: 28.8%, 15.2%, 2.8% and 3.2% *E. coli*, 250 μg/mL: 11.8% Pa, 250 μg/mL: 11.9%	Klebsiella pneumoniae-infected mice	[18]
Respiratory tract(Control hospital infections)	GO-Ag-HN	Commercially available GO	Thickness:8 nm	/	/	*P. aeruginosa* (*Pseudomonas aeruginosa*)	Decrease the survival of *P. aeruginosa*: (1) physical cutting with sharp edges. (2) enhance the penetration capacity of Ag NPs	/	/	[44]
Respiratory tract	Graphene oxide-silver nanocomposite	Modified Hummers’ method	/	/	/	*MRSA* (*Methicillin-resistant Staphylococcus aureus*)	Inhibit MRSA growth: (1) physical contact injury (2) ROS generation	30 μg/mL: 0%	/	[45]
Vagina (nanocarrier for drug delivery)	GO-PEG	Commercially available GO	1.1 × 500 nm	/	−9.47 ± 0.22 mV	*C. albicans* (*Candida albicans*)	GO-PEG promotes the death of vaginal epithelial cells infected by *C. Albicans*: (1) induce pro-apoptotic responses and damage DNA (2) induce oxidative stress (3) induce membrane potential depolarization (4) induce endoplasmic reticulum stress	/	/	[47]

## Data Availability

No new data were created or analyzed in this study. Data sharing is not applicable to this article.
